# Supplementary material for: Assessment and phenotypic identification of millet germplasm (Setaria italica L.) in Liaoning, China
Source: PeerJ. 2024 Aug 7;12:e17871. doi: 10.7717/peerj.17871 (PMC11316460; doi:10.7717/peerj.17871)
Supplement: Supplemental Information 1 [file peerj-12-17871-s001.docx]

| **Trait** | **Code numbers of 19 qualitative trait** | | | | | | | | |
| --- | --- | --- | --- | --- | --- | --- | --- | --- | --- |
|  | **1** | **2** | **3** | **4** | **5** | **6** | **7** | **8** | **9** |
| Leaf sheath color LSC | Green | Red | Purple | — | — | — | — | — | — |
| Leaf color of seedling LCS | Green | Chartreuse | Violet green | — | — | — | — | — | — |
| Bristle color BC | Yellow | Green | Purple | — | — | — | — | — | — |
| Bristle length BL | Exremly short | Short | Long | Exremly long | — | — | — | — | — |
| Protecting glume color PGC | Chartreuse | Green | Purple | — | — | — | — | — | — |
| Stigma color STC | White | Yellow | Purple | — | — | — | — | — | — |
| Anther color AC | White | Yellow | Orange | — | — | — | — | — | — |
| Seed color SC | White | Yellow | Orange | Red | Cyan | Brown | Black |  |  |
| Kernel color KC | White | Buff | Yellow | Pewter | — | — | — | — | — |
| Seedling leaf attitude SLA | Upthrow | Semi-upthrow | Flat | Downthrow | — | — | — | — | — |
| Blooming leaf attitude BLA | Upthrow | Semi-upthrow | Flat | Downthrow | — | — | — | — | — |
| Tiller habit TH | Weak | Medium | Strong | — | — | — | — | — | — |
| Branch habit BH | Weak | Medium | Strong | — | — | — | — | — | — |
| Peduncle shape PS | Upright | Semi-bent | Bent | Bow down | — | — | — | — | — |
| Ear compactness EC | Loose | Medium | Tight |  | — | — | — | — | — |
| Spike density SD | Sparse | Semi-sparse | Semi-compact | Compact | — | — | — | — | — |
| Ear shape ES | Chicken beak | Spindle | Cylindrical | Stick | Duckbill | Cat claw | Buddha hand |  |  |
| Shattering habit SH | Weak | Medium | Strong | Extremly strong | — | — | — | — | — |
| Lodging resistance LR | Extremly strong | — | Strong | — | Medium | — | Weak | — | Extremly weak |

**Table S1: Phenotype code numbers of 19 qualitative trait**
