# Supplementary material for: Assessment and phenotypic identification of millet germplasm (Setaria italica L.) in Liaoning, China
Source: PeerJ. 2024 Aug 7;12:e17871. doi: 10.7717/peerj.17871 (PMC11316460; doi:10.7717/peerj.17871)
Supplement: Supplemental Information 2 [file peerj-12-17871-s002.docx]

**Table S2: Correlation analysis of 12 quantitative traits**



| **Correlation Coefficient** | LSC | LCS | SLA | BLA | TH | BH | PS | EC | SD | ES | BL | BC | PGC | STC | AC | SC | SH | GC | LR |
| --- | --- | --- | --- | --- | --- | --- | --- | --- | --- | --- | --- | --- | --- | --- | --- | --- | --- | --- | --- |
| Leaf sheath color LSC | 1 |  |  |  |  |  |  |  |  |  |  |  |  |  |  |  |  |  |  |
| Leaf color of seedling LCS | 0.128 5 | 1 |  |  |  |  |  |  |  |  |  |  |  |  |  |  |  |  |  |
| Seedling leaf attitude SLA | -0.120 5 | 0.278 0^**^ | 1 |  |  |  |  |  |  |  |  |  |  |  |  |  |  |  |  |
| Blooming leaf attitude BLA | 0.127 1 | 0.121 5 | -0.068 9 | 1 |  |  |  |  |  |  |  |  |  |  |  |  |  |  |  |
| Tiller habit TH | -0.022 4 | -0.102 6 | -0.208 1^*^ | 0.110 9 | 1 |  |  |  |  |  |  |  |  |  |  |  |  |  |  |
| Branch habit BH | 0.002 1 | -0.107 1 | -0.216 6^*^ | 0.046 8 | 0.902 7^**^ | 1 |  |  |  |  |  |  |  |  |  |  |  |  |  |
| Peduncle shape PS | 0.077 4 | 0.022 4 | 0.013 7 | -0.071 5 | -0.056 4 | -0.055 7 | 1 |  |  |  |  |  |  |  |  |  |  |  |  |
| Ear compactness EC | -0.194 1^*^ | 0.158 8 | 0.191 0 | -0.180 1 | -0.087 9 | -0.022 9 | 0.059 6 | 1 |  |  |  |  |  |  |  |  |  |  |  |
| Spike density SD | -0.006 9 | 0.022 1 | -0.010 8 | 0.088 6 | 0.175 6 | 0.198 3^*^ | 0.011 5 | 0.132 7 | 1 |  |  |  |  |  |  |  |  |  |  |
| Ear shape ES | -0.06 1 | -0.080 7 | 0.119 6 | -0.112 1 | 0.189 2 | 0.248 4^*^ | -0.159 5 | 0.095 9 | 0.054 6 | 1 |  |  |  |  |  |  |  |  |  |
| Bristle length BL | -0.129 1 | 0.016 0 | 0.159 5 | 0.015 3 | -0.084 6 | -0.114 2 | 0.141 5 | 0.205 5^*^ | -0.156 0 | -0.118 4 | 1 |  |  |  |  |  |  |  |  |
| Bristle color BC | -0.177 0 | 0.075 2 | 0.000 0 | -0.063 6 | 0.000 0 | 0.000 0 | 0.078 3 | 0.257 8^**^ | 0.000 0 | -0.096 0 | -0.093 0 | 1 |  |  |  |  |  |  |  |
| Protecting glume color PGC | 0.128 5 | 0.004 3 | -0.052 4 | 0.198 1^*^ | 0.057 0 | -0.026 8 | 0.069 6 | 0.003 7 | 0.221 3^*^ | -0.042 2 | -0.151 9 | 0.075 2 | 1 |  |  |  |  |  |  |
| Stigma color STC | 0.019 1 | 0.185 5 | 0.112 9 | 0.080 6 | -0.193 0^*^ | -0.245 6^*^ | -0.131 4 | 0.049 5 | -0.078 1 | -0.103 6 | -0.022 6 | -0.053 1 | -0.133 8 | 1 |  |  |  |  |  |
| Anther color AC | -0.101 4 | -0.007 6 | 0.141 1 | -0.158 2 | -0.206 2^*^ | -0.271 6^**^ | -0.060 4 | 0.061 9 | -0.156 2 | -0.078 5 | 0.169 3 | -0.033 2 | -0.207 3^*^ | 0.658 8 | 1 |  |  |  |  |
| Seed color SC | 0.059 8 | -0.040 1 | -0.075 6 | -0.014 7 | 0.137 9 | 0.137 0 | 0.010 4 | -0.034 4 | -0.082 4 | -0.037 6 | -0.044 6 | -0.140 0 | -0.040 1 | 0.056 6 | 0.070 8 | 1 |  |  |  |
| Shattering habit SH | 0.107 0 | -0.0811 | -0.135 1 | 0.322 9^**^ | 0.299 5^**^ | 0.258 8^**^ | 0.067 0 | -0.069 5 | 0.350 3^**^ | 0.179 0 | 0.055 0 | -0.048 8 | 0.153 8 | -0.134 2 | -0.193 7^*^ | -0.174 3 | 1 |  |  |
| Grain color GC | -0.073 8 | -0.040 9 | 0.053 5 | 0.094 0 | 0.216 4^*^ | 0.216 0^*^ | -0.101 2 | -0.035 1 | 0.073 5 | 0.071 4 | -0.045 5 | 0.000 0 | -0.040 9 | 0.057 7 | -0.022 6 | 0.580 5^**^ | 0.100 9 | 1 |  |
| Lodging resistance LR | 0.114 3 | 0.013 2 | -0.161 2 | 0.2278^**^ | 0.157 6 | 0.146 9 | 0.110 3 | -0.261 0^**^ | 0.213 7^*^ | 0.107 1 | -0.196 5^*^ | -0.099 1 | 0.251 6^**^ | -0.243 0^*^ | -0.409 0^**^ | -0.049 3 | 0.317 9^**^ | -0.125 7 | 1 |
| Ear weight per plant EWPg | 0.061 2 | -0.094 3 | -0.326 6^**^ | 0.191 0 | 0.207 6^*^ | 0.238 2^*^ | 0.063 1 | -0.132 6 | 0.257 9^**^ | 0.012 8 | -0.006 5 | 0.063 9 | 0.097 6 | -0.219 7^*^ | -0.208 2^*^ | -0.149 8 | 0.416 2^**^ | -0.127 4 | 0.198 6^*^ |
| Seed weight per plant SWPg | 0.019 6 | -0.072 2 | -0.311 1^**^ | 0.217 3 | 0.061 5 | 0.124 0 | 0.087 8 | -0.085 3 | 0.179 8 | -0.037 1 | 0.022 4 | 0.033 4 | -0.001 0 | -0.130 4 | -0.131 4 | -0.124 2 | 0.267 4^**^ | -0.087 5 | 0.010 4 |
| Grass weight per plant GWPg | -0.137 4 | -0.078 1 | 0.030 8^**^ | -0.156 8 | 0.000 9 | -0.034 2 | -0.001 5 | 0.141 1 | -0.173 9 | 0.022 7 | 0.291 7^**^ | -0.044 3 | 0.060 1 | -0.021 5 | 0.196 7^*^ | -0.125 1 | -0.092 0 | -0.124 9 | -0.272 7 |

Note: r_0.05_=0.1918，r_0.01_=0.2504
